# Co-Culturing *Bacillus* Strains for Enhanced Antimicrobial Activity

**DOI:** 10.3390/antibiotics14090908

**Published:** 2025-09-08

**Authors:** Charlie Tran, Russell S. Addison, Ian E. Cock, Xiaojing Chen, Yunjiang Feng

**Affiliations:** 1 Institute of Biomedicines and Glycomics (IBG), Griffith University, Nathan, QLD 4111, Australia; charlie.tran@griffithuni.edu.au (C.T.); r.addison@griffith.edu.au (R.S.A.); 2 School of Environment and Science, Griffith University, Brisbane, QLD 4111, Australia; i.cock@griffith.edu.au; 3 Bioproton Pty Ltd., Acacia Ridge, QLD 4110, Australia; wendy@bioproton.com

**Keywords:** antimicrobials, *Bacillus*, co-culture

## Abstract

**Background/Objectives**: *Bacillus* probiotic mixtures have been used as a novel source of antimicrobial production. However, there is a need to emphasize the potential of co-cultured *Bacillus* strains and to understand the underlying mechanisms involved in their combination formulation. **Methods**: *Bacillus* strains BPR-11, BPR-16, and BPR-17, were cultured either as mono or multi-cultures. The bacterial growth and density were evaluated by measuring their optical density. The chemical profiles of their bioactive extracts were analysed by LC-MS, and their antimicrobial activity were tested against selected pathogens. **Results**: Our results reveal that co-cultured strains significantly increased bacterial growth, with an optical density level of, 2.67 which was significantly higher than the monocultures of BPR-11 (2.24), BPR-16 (2.32), and BPR-17 (2.22). Furthermore, the ethyl acetate extracts from these bacterial cultures showed that the combined co-culture (F1) exhibited the highest antimicrobial activity, with MICs of 25 µg/mL against *C. perfringens*, *E. coli*, and *S. aureus*, and 50 µg/mL against *P. aeruginosa* and *S. enterica*. In contrast, the monocultures BPR-16 and BPR-17 showed moderate activity with MICs of 50 µg/mL against *C. perfringens*, *E. coli*, and *S. aureus*, and 100 µg/mL against *P. aeruginosa* and *S. enterica*. BPR-11 had the lowest antimicrobial activity, with MICs of 100 µg/mL against *C. perfringens*, *E. coli*, and *S. aureus*, and no activity against *P. aeruginosa* and *S. enterica*. Metabolite profiling showed that the extract from the co-culture had a marked increase in the production of antimicrobial metabolites, including C_13_-C_16_ surfactin C. Lastly, the metabolism study of surfactin C analogues suggested that they were highly stable (99%) when incubated with cytochromes P450 over 120 min. **Conclusions**: Together, these findings highlight the potential for multi-strain co-culturing to develop new antimicrobials and provide valuable insights into the synergistic effects for antimicrobial production.

## 1. Introduction

Co-culturing microbial strains is currently been explored for their production of novel antimicrobial compounds [1]. This technique involves fermenting multiple microbial strains together, which allows them to interact and synergize together to alter metabolite production that is unique due to these interactions [2,3,4]. Studies have shown that the co-culturing of *B. subtilis* and *B. licheniformis* increased antimicrobial activity and production of bioactive metabolites [5]. These metabolites include enzymes, peptides, and non-ribosomal compounds, which target pathogenic organisms, which can improves the breakdown of indigestible material in animal feed and reduce feed waste [2,6,7]. Moreover, co-culturing microbial strains has been shown to regulate the immune system function and promote optimal digestive health [8]. These findings underscore the potential of utilizing multi-strain formulations for the discovery and enhanced production of antimicrobials [9].

Despite the potential of multi-strain formulations, further research is needed to optimize co-culture formulations to maximize antimicrobial yield [3,10,11]. This is mainly due to the extensive selection of compatible strains currently unexplored and the complexity of synergistic microbial interactions [12]. Modern analytical chemical techniques and modern access to metabolomics instruments and databases have enabled researchers to gain insights into the chemical profile changes caused by these formulations. Additionally, spore-forming *Bacillus* microorganisms have been shown to be promising candidates for co-culture systems, due to their ability to produce a vast array of antimicrobial metabolites and their stability under harsh environmental and storage conditions [13]. These advancements have allowed researchers to map out the exact metabolic pathways and changes caused by these microbial interactions [14].

These interactions have gained significant attention due to their potential to enhance antimicrobial production [15]. These interactions can often lead to changes in metabolic exchange pathways, nutrient utilization, and even pH conditions, which affects microbial growth and metabolite production [16]. For instance, multi-strain formulations often lead to alterations in gene expression, typically through the activation of silent gene clusters [17]. These silent genes clusters, which is only activated through the stress of multiple strains, can activate the production of novel antimicrobials. However, not all silent gene clusters produce compounds even when activated. Furthermore, molecular interactions between different strains can cause inherent changes in the fermentation conditions, often leading to changes in nutrient utilization and even changes in pH conditions that affect microbial growth. These mechanisms highlight the interactions between different co-culture strains, and further research into their effects will provide insights into their complex interactions to optimize their maximize their potential and their long-term efficacy [3].

To ensure the long-term efficacy of multi-strain probiotics, it is crucial to understand the variability and stability of the produced antimicrobial metabolites. Recent studies have focused on optimizing the media conditions, fermentation conditions and gene expression to optimize antimicrobial production [18,19,20,21]. However, there is a gap in the long-term variability of these metabolites especially when it comes to metabolism studies. These studies, especially in the presence of hepatic microsomes from different hosts, can provide valuable insights to their variability once produced in the gut environment [22]. As any chemical transformation to these metabolites can significantly affect their nutritional and functional profiles, elucidating these mechanisms will allow researchers to track any changes and degradation through enzyme activity and to ensure their efficacy in formulation development.

Thus, this study aims to investigate the influence of multi-strain probiotics and their impact on growth, survival, and host functionality. We selected three *Bacillus* strains, BPR-11, BPR-16, and BPR-17, based on their previously demonstrated antimicrobial activity against pathogens and ability to produce antimicrobial compounds [23,24]. To further elucidate the interactions between these strains and their complex interactions during multi-strain fermentation, each strain was cultured both individually, and as a multi-strain culture termed F1. The resulting bacterial cultures were extracted with ethyl acetate (EtOAc), and the extracts were evaluated for their antimicrobial activity against a panel of pathogens, including *Escherichia coli* (*E. coli*), *Clostridium perfringens* (*C. perfringens*), *Pseudomonas aeruginosa* (*P. aeruginosa*), *Staphylococcus aureus* (*S. aureus*), and *Salmonella enterica* (*S. enterica*). Furthermore, this study analysed the chemical profiles of the extracts using liquid chromatography-mass spectrometry (LC-MS) to identify any changes in the production of compounds produced by the selected *Bacillus* strains. Finally, to investigate the long-term stability of these formulations, we incubated identified compounds from the co-culture fermentation with a mixture of cytochrome P450 isozymes extracted from poultry chicken livers over a 120-min period [25]. This *in vitro* method replicates the gut environment to assess the stability of these compounds.

## 2. Results

### 2.1. Fermentation of Mixed Bacillus Strains

*Bacillus* strains BPR-11, BPR-16, and BPR-17 was selected for their antimicrobial activity and benefits to poultry health [23,24]. Previously, the combination formulation of F1 containing BPR-11, BPR-16 and BPR-17 (Table 1) significantly improved the feed conversion ratio (1.49 vs. 2.10 at day 14) and body weight (847.0 g vs. 787.4 g at day 21) compared to animal feed without F1 supplementation. Additionally, F1 was shown to produce antimicrobial lipopeptides C_13_–C_16_ surfactin C, which may inhibit the growth of poultry related pathogens and subsequently increase broiler growth performance and nutrient utilization. Thus, further analysis was needed to understand their multi-strain mechanisms and their potential impact on antimicrobial metabolite production.

**Figure 1:**
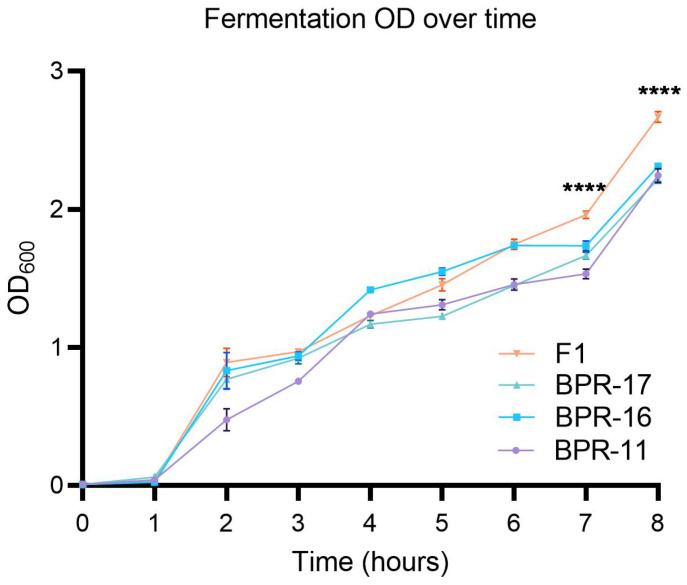
Fermentation OD_600_ over time with sigmoidal bacterial growth phases. The graph shows the optical density (OD) measurements over time for bacterial strains BPR-11, BPR-16, BPR-17, and F1. OD_600_ was monitored every 60 min for 8 h. Values represent the mean ± SD values of 3 replicates. Strain F1 exhibited the highest final OD_600_ followed by BPR-17, BPR-16 and BPR-11 respectively. The differences between F1 and individual monocultures were statistically significant. **** indicates *p* < 0.001 by one-way ANOVA followed by Tukey’s post hoc test.

To examine growth rates, each strain was monocultured alongside the multi-strain F1 in TSB medium using a 1.5 L bioreactor under controlled conditions (37 °C, 300 rpm, pH 7, O_2_ 300 ppm) for 8 h. Each strain was cultured on a plate and a single colony was taken from each plate to form four bacterial suspensions in TSB, three containing 1 × 10^8^ CFU of each individual strain, and one containing a mixture of all three strains (each at 1 × 10^8^ CFU) and was incubated for 24 h at 37 °C. Subsequently, each at 1 × 10^8^ CFU of each culture was fermented with the optical density being measured every hour at 600 nm to monitor the impact on bacterial growth and measure any significant differences in growth rates between the individual strains and F1 co-culture (Figure 1). Each of the individual BPR-11, BPR-16 and BPR-17 strains had similar growth profiles after 8 h, reaching final OD_600_ values of 2.24, 2.32, 2.22 respectively. In contrast, the F1 co-culture consistently grew faster and achieved a significantly higher final OD_600_ of 2.67, suggesting the co-culturing of BPR-11, BPR-16 and BPR-17 in F1 were favourable for bacterial proliferation compared to the monocultures. In addition, the accelerated growth of F1 suggests a beneficial interaction in-between the strains that promotes microbial growth that could potentially increase the yield of antimicrobial metabolites produced.

### 2.2. Antimicrobial Activity

The antimicrobial activity of individual strain and F1 was evaluated against five pathogens: *C. perfringens*, *E. coli*, *P. aeruginosa*, *S. aureus*, and *S. enterica* (Table 2). After fermentation, the cultures were sonicated and centrifuged to remove the cell pellet. The supernatant was divided into two equal parts. One part was extracted with ethyl acetate to obtain the EtOAc extract, while the other part was freeze-dried and resuspended in water to yield a crude extract. Both EtOAc and crude extracts were tested against the previously mentioned pathogens. Among all EtOAc extracts, co-culture of mixed strains F1 exhibited the highest antimicrobial activity, with MICs of 25 µg/mL against *C. perfringens*, *E. coli*, and *S. aureus*, and 50 µg/mL against *P. aeruginosa* and *S. enterica*. In contrast, BPR-16 and BPR-17 showed moderate activity with MICs of 50 µg/mL against *C. perfringens*, *E. coli*, and *S. aureus*, and 100 µg/mL against *P. aeruginosa* and *S. enterica*. Strain BPR-11 had the lowest antimicrobial activity, with MICs of 100 µg/mL against *C. perfringens*, *E. coli*, and *S. aureus*, and no activity against *P. aeruginosa* and *S. enterica*. Notably, the crude extracts of all strains (BPR-11, BPR-16, BPR-17, and F1) were inactive against all of the pathogens tested.

### 2.3. Metabolite Profiling by HPLC

**Figure 2:**
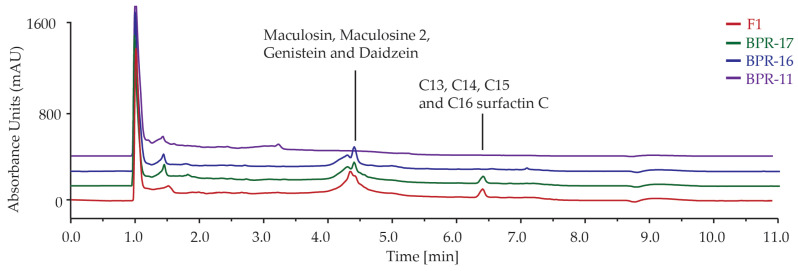
HPLC Chromatograms of EtOAc extracts of bacterial strains BPR-17, BPR-16, BPR-11, and F1. The peaks between 1–2 min were associated with the metabolites from the culture medium. Peaks at 4–5 min represent maculosin, maculosine 2, genistein and daidzein, while peaks at 6–7 min represent C_13_, C_14_, C_15_ and C_16_ surfactin C.

The metabolites produced by strains BPR-11, BPR-16, BPR-17 and co-culture F1 were analysed by UHPLC (Figure 2). The analysis showed that the EtOAC extracts from BPR-11, BPR-16, BPR-17 and F1 had similar chemical profiles, with metabolites eluting at 4–5 min and 6–7 min. Our previous study showed that the peaks between 1–2 min were associated with the metabolites from the culture medium, while the peaks at 4–5 min represent maculosin, maculosine 2, genistein, and daidzein, and the latter peaks at 6–7 min containing C_13_-C_16_ surfactin C (Figure 2) [23]. Although our findings did not reveal a novel peak due to multi-strain fermentation, the peak intensities in the F1 chromatogram were higher than those in the monoculture BPR-17, BPR-16 and BPR-11. As the previous study revealed that the surfactin C exhibited both broad and enhanced antimicrobial activity, this suggests that the multi-strain formulation may have led to an increase in metabolite production compared to its mono-culture counterparts [23].

### 2.4. Quantification of Surfactin C Metabolites

**Figure 3:**
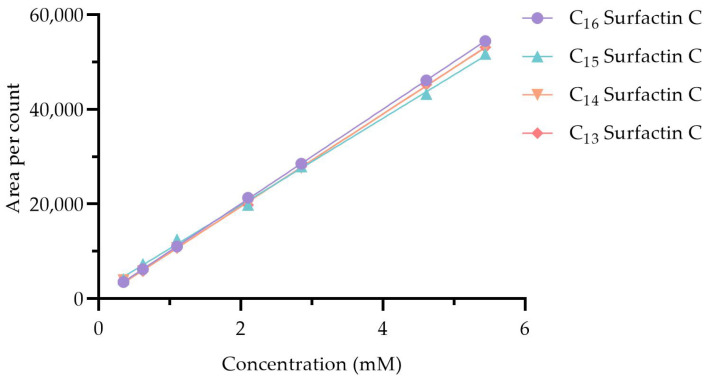
Standard concentration curve of pure C_13_, C_14_, C_15_ and C_16_ surfactin C by LC-MS. The measured R^2^ values measured were with C_13_ surfactin C (0.9906), C_14_ surfactin C (0.9994), C_15_ surfactin C (0.9984) and C_16_ surfactin C (0.9893). These R^2^ values were approximately 0.99, highlighting the linearity between the measured concentration and response.

Before the surfactin C metabolites could be quantified, the instrument response to pure surfactin C analogues needed to be validated by constructing a response curve. Eight serial dilutions (0.01–10 mM) were prepared from a 10 mM stock solution, which was subsequently injected onto LC-MS to ensure that the instrument response was proportional to the analyte concentration (Figure 3). A C_18_ column was employed with a gradient elution program consisting of water and acetonitrile, both containing 0.1% formic acid. The gradient was carefully optimized to enhance resolution, sensitivity, and minimize co-elution of surfactin C analogues with other metabolites. Further analysis of the acquired LC-MS was performed by calculating the peak areas of the selected surfactin C compounds using genesis peak detection. The area was then plotted against the concentration, and the line of best fit was constructed to reveal an R^2^ value indicating measured predictability. The line of best fit between all samples showed an R^2^ between 98–99% in all compounds as indicated in Figure 3, highlighting a consistent trend of area response to analyte concentration.

**Figure 4:**
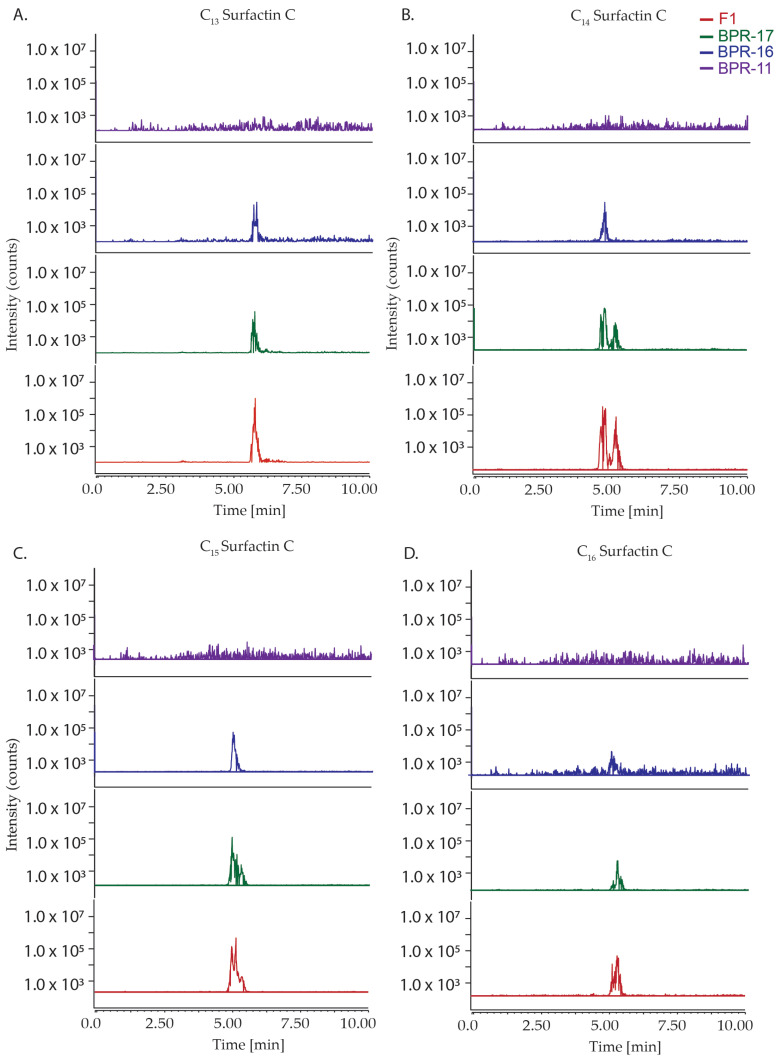
Representative chromatograms displaying the production of C_13_ (**A**), C_14_ (**B**), C_15_ (**C**), and C_16_ (**D**) surfactin homologues produced by *Bacillus* strains BPR-11, BPR-16, BPR-17, and their multistrain-culture F1. These results are consistent across multiple analyses. The highest peak intensities were consistently observed in F1 throughout each spectrum, with lesser amounts detected for BPR-17 and BPR-16. BPR-11 consistently showed little to no detection of the surfactin homologues across repeated experiments.

After the response curve was constructed, the fermentation media was analysed by LC-MS to quantify the production of the surfactin C metabolites. To achieve optimal separation and accurate quantification, the same C_18_ column was employed using the same gradient elution program consisting of water and acetonitrile, both containing 0.1% formic acid water to ensure the comparison of the experiment to the calibration curve. A total of four selected ion monitoring (SIM) channels were extracted from the crude fermentation samples, which allowed for the quantification of C_13_-C_16_ surfactin C (Table 3, Figure 4).

The SIM for each surfactin C analogue revealed differences in the production of these metabolites amongst the strains. For BPR-11, there was no observed production of C_13_-C_16_ surfactin C while BPR-16 and BPR-17 produced observable peaks, which may be associated with its measured antimicrobial activity. The co-culture F1 had more pronounced peaks compared to the rest of the mono-culture samples, suggesting that the multi-strain fermentation of these strains enhanced the production of C_13_-C_16_ surfactin C compared with the individual mono-culture strains. This analysis may be related to the increased antimicrobial activity compared to the mono-culture strains, with further quantification needed to measure its exact impact.

**Figure 5:**
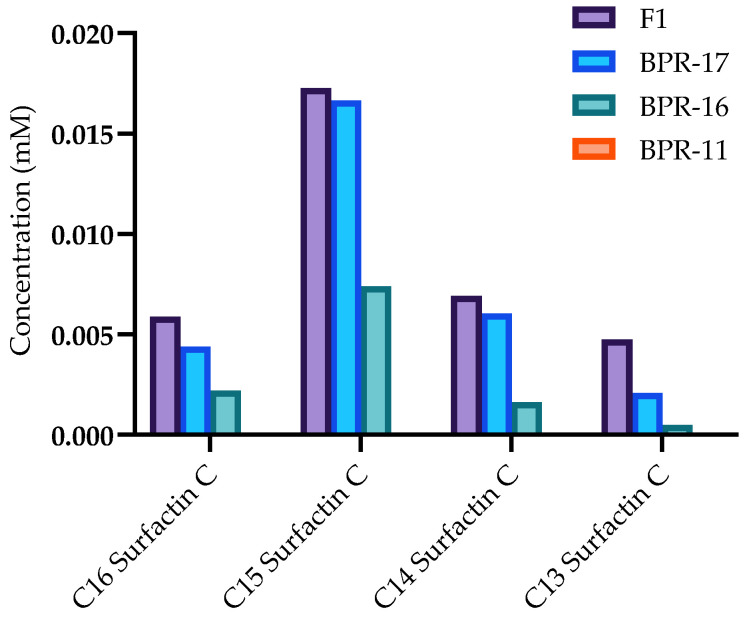
Quantification of surfactin C analogues in co-culture and monocultures. The bar graph illustrates the quantification of C_13_-C_16_ surfactin C produced by the co-cultured *Bacillus* strain F1 and individual mono-cultured strains BPR-17, BPR-16, and BPR-11. The data reveals significant variations in surfactin C production among the strains, with F1 exhibiting the highest levels across all analogues, followed by BPR-17, BPR-16, and BPR-11.

The comparison and integration of the surfactin C peak areas compared to the constructed response curve reinforced the heightened production in F1 (Figure 5). Of crucial note, the highest level of surfactin C analogues was present in F1 in comparison to the monocultures, emphasizing the benefits of co-culturing strains compared to its mono-culture counterparts. Interestingly, the quantification experiment showed an elevated level of C_15_ in comparison to C_16_, C_14_ and C_13_ levels respectively, suggesting C_15_ surfactin C as the major analogue present in the sample.

### 2.5. Surfactin C Stability Study

After determining the presence of surfactin C analogues in F1, BPR-17 and BPR-16, an in vitro metabolism assay was conducted to investigate the bioavailability and stability of these compounds. This study involved incubating the bioactive metabolites with liver microsomes obtained from chickens to predict and evaluate their potential efficacy and bioavailability once digested [27]. The microsomal assay was performed by first harvesting the chicken microsomes from the liver. Once obtained, these were incubated with the surfactin C compounds in the presence of NADPH, which serves as an essential cofactor for cytochrome P450 enzymes [28]. The degradation of surfactin C analogues over time was monitored by measuring their peak areas using LC-MS/MS [29]. Additionally, a positive control compound (verapamil) was used to confirm the metabolic activity of the liver microsomes.

**Figure 6:**
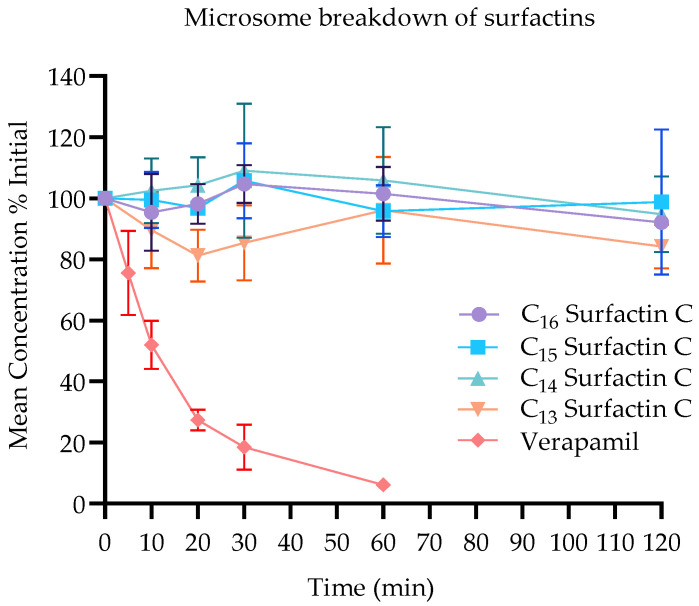
Metabolic stability of C13-C16 surfactin C analogues in chicken liver microsomes over 120 min. Data points represent mean ± standard deviation (*n* = 3 independent replicates). The slowly decreasing lines indicate minimal degradation of the surfactin C analogues over the incubation period, suggestive of high metabolic stability within the gut. No statistically significant differences were observed between time points for any analogue (*p* > 0.05, one-way ANOVA with Tukey’s post hoc test). Quantification was performed using LC-MS/MS analysis, with results expressed as percentage of initial concentration remaining.

The results showed minimal to no decrease in surfactin C concentrations after 120 min of incubation with the microsomes (Figure 6). Specifically, the concentrations of C_13_, C_14_, C_15_ and C_16_ surfactin C remained stable with approximately 84%, 95%, 99%, and 92% of their initial values, respectively, remaining after the incubation period. The metabolic stability of surfactin C was further evaluated by their half-life (t_1/2_), with the t_1/2_ value calculated using the equation t_1/2_ = ln (2)/k, where k is the elimination rate constant derived from the slope of the natural log-transformed concentration versus time plot (Table 4). The t_1/2_ for all surfactin C analogues was found to be >120 min, indicating high metabolic stability and a favourable pharmacokinetic profile. These findings indicate a low susceptibility of surfactin C analogues to hepatic enzymatic degradation, highlighting their potential as a stable and effective animal feed supplement.

## 3. Discussion

This study investigated the effects of co-culturing F1 strains compared to their monocultures on microbial growth, antimicrobial activity and metabolite production. Our findings showed that the multi-strain formulation F1 increased microbial growth, antimicrobial activity and enhanced production of surfactin C analogues, which exhibited high stability and resilience against breakdown by poultry P450 enzymes. These findings highlight the advantages of employing multi-strain formulations and their long-term stability in agricultural applications.

### 3.1. Elevated Microorganism Growth

The present study showed that co-culture F1 strains resulted in elevated microbial growth compared to individual monocultures [30]. This finding is consistent with previous studies that have reported microbial growth enhancements in multi-strain formulations [31]. The synergistic interactions between different strains during co-culture may contribute to this elevated growth through various mechanisms, such as the production of growth promoting factors, signalling molecules and the formation of synergistic biofilm structures [30].

Previous research has shown that *Bacillus* may release signalling molecules, which can subsequently trigger the quorum sensing mechanisms needed for these microorganisms to form biofilm structures [32]. These biofilms allow microorganisms to better coordinate their gene expression and increasing their resilience to external environmental stressors. Additionally, multi-strain formulation may have produced multiple enzymes, organic acids (e.g., acetic acid, lactic acid) and vitamins (e.g., biotin, thiamine) that affect the final chemical profile of the fermentation and provide an additional sources of nitrogen and carbon to be utilized by other complementary strains [30]. Lastly, the enzyme production from these varied microbial strains may breakdown the indigestible material within the fermentation media, thus providing an additional source of nutrients for the growth of complementary strains [33].

While the synergy via co-factors and biofilms is plausible, future research should employ enzyme assays and cellular studies to confirm these mechanisms, with a particular emphasis on using efficient biofilm detection techniques. This approach would provide valuable insights into the molecular and cellular processes involved in multi-strain interactions. Additionally, further exploration and optimization of multi-strain fermentation starting formulations would help validate their impacts and assist in maximizing the synergistic interactions between these multi-strain formulations for use in agricultural practices [34]. By combining advanced biofilm detection methods with detailed mechanistic studies, researchers can gain a more comprehensive understanding of how these microbial communities function and interact in agricultural settings.

### 3.2. Increase Production of Antimicrobial Metabolites

Following the elevated microbial fermentation growth, subsequent antimicrobial bioassay experiments showed that the co-cultured F1 had an elevated antimicrobial activity compared to the monocultures. These findings suggest that the multi-strain interactions in F1 may have triggered the activation of silent genes, horizontal gene transfer, or the formation of synergistic biofilm structures, leading to an increased production of bioactive compounds [35].

The activation of several mechanisms, such as the activation of silent genes due to external stresses has been previously reported as a potential mechanism to enhance antimicrobial production [36]. In the case of F1, the co-culture of similar *Bacillus* species may have created a unique environment that induced the expression of genes responsible to produce surfactin C analogues. However, the specific mechanisms underlying these synergistic effects remains speculative at this stage and further investigation is required. These could include metabolomic profiling to identify changes in metabolite production, as well as co-culturing experiments with specific pathway inhibitors to elucidate the contribution of individual pathways to the observed synergistic effects.

The increased production of antimicrobial surfactin C analogues by the co-cultured F1 formulation highlights the potential of multi-strain interactions for enhancing the production of bioactive compounds. While this study did not detect any novel peaks indicative of a novel bioactive compound, optimized fermentation, increased fermentation volumes, and more precise analytical tools could potentially reveal the presence of minor compounds unique to the fermentation of the multi-strain F1 formulation. Additionally, inconsistencies in the antimicrobial activity for mono strains were observed between the current study and previous findings [23]. Fermentation parameters including the differences in the time incubated (8 h vs. 6 h), state when harvesting (late log vs. stationary phase) or subtle factors not accounted for such as bacterial state when inoculated (spore vs. vegetative). To mitigate this inconsistency and ensure reliability, future experiments should either clarify or control these conditions and their effects on antimicrobial activity and metabolite production [37]. As *Bacillus* species are typically facultative aerobes, future research should investigate how these factors influence the mechanisms and production of antimicrobial compounds by these microorganisms. Lastly, experimental conditions used in this study may not accurately reflect the growth conditions in the chicken gut, which are characterized by low pH and microaerophilic conditions. Hence, further testing the effects of F1 on beneficial gut bacteria is crucial to ensure that the treatment selectively targets pathogens while preserving a healthy gut microbiome.

### 3.3. Stability Experiment

This observed stability of sufactin C analogues aligns with previous findings in the literature, which have reported the resilience of surfactins to proteolytic cleavage and their stability under various temperature and pH conditions [38].

The stability of surfactin C analogues in the presence of liver microsomes has important implications for their potential application as antimicrobial agents in agriculture and human health [39]. Previous studies have shown that surfactins exhibit a broad antimicrobial spectrum against bacteria by interfering with the structural integrity of the cell wall, leading to dysfunction and cell leakage [40]. The low susceptibility of surfactins to oxidative metabolism by cytochrome P450 enzymes, as demonstrated in this study and previously reported with microsomes from other animals [41], further supports their potential for long-term viability and inherent safety [42].

This measured stability of the surfactin C analogues reflects previous observed findings in the literature. This observed stability has been previously reported with microsomes from other animals including humans, mice, and rats, and has been attributed to its low susceptibility to oxidative metabolism by the cytochrome P450 enzymes abundant in liver microsomes [43].

However, to fully understand the complex interactions between F1 and the host strain, as well as to optimize multi-strain interactions for effective antimicrobial production, further research is needed. Investigating the metabolite profile in other measurable fluid systems, such as blood and saliva, could provide valuable insights into the activated host pathways and contribute to the development of novel antimicrobial strategies [41,44]. Furthermore, research is needed to understand the exact interactions between F1 and the metabolite profile in other measurable fluid systems, such as blood and saliva, to elucidate the complex interactions and optimize multi-strain interactions for effective antimicrobial production [39].

### 3.4. Limitations

While this study provides valuable insights into the interactions between the multi-strain formulation of F1 and its influence to heighten the production of surfactin C analogues, further research is necessary to normalize and optimize its fermentation conditions. The observed inconsistencies in the performance of strain BPR-11 warrant further investigation, with previous research showing a MIC of 25 μg/mL against *C. perfringens*, *E. coli*, and *S. aureus* at 25 μg/mL and the MICs being 100 μg/mL. While the fermentation conditions and extraction methods were consistent between the two chapters, variability in BPR-11’s may be inherent and attributed by other factors. These include the differences in the time incubated (8 h vs. 6 h), state when harvesting (late log vs. stationary phase) or subtle factors not accounted for such as state of bacterial state when inoculated (spore vs. vegetative). To mitigate this inconsistency and ensure reliability, future experiments focusing on the reproducibility of BPR-11’s performance and the factors influencing its metabolite production could help explain the differences [37]. Furthermore, metabolite profiling by HPLC of the crude (non-active) extract is necessary to compare the differences with the active ethyl acetate extracts. This analysis would provide valuable insights into the observed differences between both cultures and to further support our findings for the heightened production of bioactive metabolites [43]. Lastly, further studies should focus on the exact pathways activated due to microbial supplementation, to help reveal their impact on the post and its possible interaction with the surrounding microbiota and physiological systems to further develop its use as a agriculture strategy [44].

## 4. Materials and Methods

### 4.1. General Experimental Procedure

Tryptone Soya broth (TSB), Mueller-Hinton broth (MHB), Tryptone Soy Agar (TSA), and Mueller-Hinton Agar (MHA) were sourced from Oxoid Australia (Thebarton, Australia). Indicator strains *C. perfringens* (ATCC 13124), *E. coli* (ATCC 43887), *P. aeruginosa* (ATCC 10145), *S. aureus* (ATCC 6538), and *S. enterica* (ATCC 6960), were obtained from Invitrogen (Melbourne, Australia). All strains except *C.perfringens* were grown in MHB or on MHA plates. *C. perfringens* was initially grown in blood agar under anaerobic conditions using an airtight sealed plate and was subsequently grown in liquid medium using MHB. All strains were subsequently grown under aerobic conditions at 37 °C for 18–24 h. Anti-foam C (polydiethylsiloxane) and resazurin were acquired from Sigma Aldrich (Melbourne, Australia). Solvents used for extraction and purification were of HPLC grade, sourced from RCl Labscan (Gillman, Australia). Water was deionized and filtered through a Millipore Milli-Q PF system (0.45 µm).

### 4.2. Bacillus Strains and Fermentation

Three *Bacillus* strains (BPR-11, BPR-16, and BPR-17) were provided by Bioproton Pty Ltd. (Brisbane, Australia). These strains were initially streaked onto TSA plates (1.5% agar) and incubated for 16 h. A single colony from each plate was then isolated and four bacterial suspensions were prepared in TSB (Oxoid, Basingstoke, UK) (50 mL), three containing 1 × 10^8^ CFU of each individual strain, and one containing a mixture of all three strains (1 × 10^8^ CFU each strain) and were incubated with vigorous shaking (300 rpm) for 24 h at 37 °C. For subsequent large-scale fermentation, 1 × 10^8^ CFU of each individual strain was taken from each suspension and were injected into a 1.5 L bioreactor containing TSB (1 L) for 8 h at 37 °C. The fermentations underwent continuous agitation at 300 rpm, while maintaining the pH at 7 and O_2_ at 300 ppm. To control foaming, antifoam C was added to the bioreactor and the OD_600_ value was measured every hour using a Jasco V-650 spectrophotometer (Jasco, Tokyo, Japan) at 600 nm. The fermented cultures were subsequently stored at −20 °C until they were processed for extraction.

### 4.3. Extraction

The culture broth (1 L) was subjected to sonication in three 5-min intervals, followed by centrifugation at 8000 rpm at 4 °C. The resulting cell pellet was collected and separated from the broth. Aliquots of the broth (200 mL) were extracted with an equal volume of ethyl acetate 3 times. The combined EtOAc extracts were then evaporated using a rotatory evaporator and concentrated to dryness using a freeze drier to yield an EtOAc extract. The remaining 200 mL portion of the H_2_O broth was freeze dried and then resuspended in water to obtain the crude extract.

### 4.4. Antimicrobial Assay

Bacterial strains were streaked onto MHA plates (28 g/L, 3% agar) and left overnight for 16 hrs. A single colony was then swabbed into 10 mL of MHB broth and incubated at 37 °C until an optical density (OD) of 0.1 at 600 nm was achieved. To assess antimicrobial activity, 78 µL of the bacterial broth was added to wells containing 2 µL of either the crude extract (800, 400, 200, and 100 µg/mL) or EtOAc extract (100, 50, 25, and 12.5 µg/mL) in a 96-well plate. The 96-well plates were incubated for 16 h overnight at 37 °C. Subsequently, 25 µL of resazurin dye (0.01% *w*/*v*) was then added to each well and incubated for an additional 6 h at 37 °C. The antimicrobial activity was quantified by measuring the resazurin reduction at 525/580 nm using a Biotek Spectre 2 microplate reader. Serial dilutions were prepared in DMSO. Gentamicin (Sigma-Aldrich) served as the positive control, while DMSO was used as the negative control. Each concentration was tested in triplicate (*n* = 3) and a growth control without any extract or antibiotic was included to ensure proper growth of organisms.

### 4.5. Chemical Profile by LC Analysis

Initial chemical profiling of the *Bacillus* EtOAc extracts was performed using a Thermo Scientific UltiMate 3000 HPLC system. Chromatographic separation was achieved using an Onyx Monolithic C18 column (100 × 3.0 mm). The mobile phase consisted of (A) water and (B) methanol, both containing 0.1% trifluoroacetic acid (TFA). A gradient elution was employed, starting with 90% A and 10% B and increased to 100% B over 6.5 min, followed by a 2.5-min hold at 100% B. The gradient was then reduced to 10% B within one minute and maintained at this concentration for an additional 2 min to equilibrate the column. The flow rate was set at 4.0 mL/min, and the injection volume was 5 μL (5 mg/mL). Data acquisition and processing were carried out using Chromeleon software (version 7.2.1). The chromatogram peaks were identified as metabolites from the culture medium by comparing their retention times and mass-spectra with those of pure standards, consistent with previous studies [23].

### 4.6. Surfactin C Quantification by LC-MS Analysis

A calibration curve of pure surfactin C analogues was constructed by preparing eight serial dilutions from a 10 mM stock solution that was subsequently analysed by UHPLC-MS. The area under the curve obtained in the acquired spectra was measured and plotted against the concentration to obtain a response curve. The UHPLC-MS instrument utilised was a ThermoScientific UltiMate 3000 HPLC system coupled to a Thermo Scientific MSQ Plus single quadrupole mass spectrometer (Thermo Fisher Scientific, Waltham, MA, USA) with an electrospray ionization (ESI) source. The mobile phase consisted of (A) water with 0.1% formic acid and (B) methanol with 0.1% formic acid with gradient elution of 5% (A) to 0% (A) in 12 min. The gradient was dropped to 10% B over 0.1 min and maintained there for 1 min. The flow rate was set at 0.3 mL/min, and the injection volume was 5 μL. Chromeleon version 7.3.2 was used for data acquisition and processing.

A 50 mL volume of fermented broth of single and co-culture was dried using an air blower and subsequently freeze-dried. The extract was dissolved in MeOH (975 µL) and cyclosporin A (10 μM, 25 µL) added as an internal standard. A 10 µL volume of the supernatant was analysed using UHPLC-MS using an Accucore C_18_ column (2.6 μm, 150 × 2.1 mm) maintained at 40 °C. The profile was compared with the retention time and mass-spectrum of the pure standard to confirm the presence of the test compounds. The area of surfactin C analogue in the samples was integrated against the calibration curve of the pure samples to determine the concentration of the compound based on the peak area. The mass spectrometer was operated in negative ionisation mode with a scan range of 100–1500 *m*/*z* with selected ion monitoring channel turned on.

### 4.7. Microsome Preparation

Preparation of microsomes from liver tissue was performed according to the protocols developed by Knights et al. 2016 [45]. Livers from chickens approximately 42 days old were obtained from Golden Cockerel poultry processors (Mount Cotton, QLD, Australia). Immediately after harvesting, the livers were washed in 1.15% KCl in lab grade H_2_O to remove excess blood and transported to the laboratory on ice for processing. To prepare the microsomes, 10 g of liver tissue was homogenized in buffer (10 mM potassium phosphate, pH 7.4) using an Ultraturrax-style homogeniser with three 30-s bursts, cooling the sample on ice between each burst. The homogenate was then centrifuged at 7000× *g* for 10 min at 4 °C. The crude pellet was discarded, and the supernatant was further separated by ultracentrifugation at 105,000× *g* for 60 min at 4 °C. The supernatant was decanted, and the pellet was rinsed with 1 mL of microsome buffer. The rinsing and ultracentrifugation process was repeated twice, and the resulting pellet was resuspended in microsome storage buffer and stored at −80 °C until further use. A Bradford assay was used to determine the concentration of the protein in the sample [46].

### 4.8. Surfactin C Metabolism Study

The test compounds C_13_-C1_6_ surfactin C (10.0 mM) were prepared in DMSO, and the metabolic stability of test compounds was determined in triplicate in an oscillating water bath (Grant) at 37 °C and 150 RPM [25]. Positive control (verapamil; 0.001 mM) and negative control (without NADPH regeneration system) incubations were conducted concurrently. Tubes containing potassium phosphate buffer (100 mM) at pH 7.4, microsomal protein stock solution (0.5 mg/mL), test compound solution (1.0 µM) and glucose-6-phosphate dehydrogenase (1 IU/mL) were preincubated at 37 °C for 5 min prior to the reaction being initiated by the addition of 25 µL of a 20× stock of an NADPH regeneration system (NRS) containing NADP (1.3 mM). The total incubation volume was 0.5 mL. The mixture was then incubated at 37 °C in an oscillating water bath (Grant) at 150 RPM for 0, 10, 20, 30, 60, and 120 min. At each time point, reactions were terminated by the addition of 150 µL of ice-cold acetonitrile containing cyclosporin A (60 ng/mL) as an internal standard for test compounds or clotrimazole (50 ng/mL) for verapamil. The test tubes were centrifuged at 13,000× *g* for 5 min, and ~100 µL of supernatant was transferred to polypropylene 96-well plates for LC-MS/MS analysis using a Waters ALLIANCE HPLC system coupled to a Waters Quattro Micro triple quadrupole mass spectrometer (Milford, MA, USA). Chromatographic separation was achieved using a Phenomenex HPLC C18 column (4.6 × 50 mm, 3 µm). Mass spectrometric detection was performed in negative ion multiple reaction monitoring mode with argon as the collision gas. Data acquisition and processing were carried out using MassLynx software (version 4.1, Waters). The half-life (t1/2) of each test compound was calculated using the equation t_1/2_ = ln(2)/k, where k is the elimination rate constant derived from the slope obtained from the ln-concentration versus time plot.

## 5. Conclusions

In conclusion, this experiment showed the potential of utilising multi strain F1 co-culture in comparison to its monoculture counterparts. F1 stimulated microbial growth and antimicrobial activity against several agricultural-related strains such as *E. coli*, *C. perfringens*, and *S. enterica*. Furthermore, the quantification experiments highlighted the elevated production of stable bioactive C_13_-C_16_ surfactin C compounds that have been reported for their antimicrobial and immunomodulatory effects. The enhanced production of antimicrobial surfactin C alongside its stability in the presence of poultry microsomes suggests its potential as animal feed supplement. Further investigations into the molecular mechanisms underlying its synergistic interactions in multi-strain formulations will provide valuable insights into developing optimal strategies for strain selection and co-culture techniques in antimicrobial discovery and production.

## Figures and Tables

**Table 1:** *Bacillus* strains, origins and CBS numbers.

Strains	Strain Type	Origin	CBS Number *
BPR-11	*B. amyloliquefaciens*	Soil and vegetation	141,692
BPR-16	*B. amyloliquefaciens* **	Soil and vegetation	148,295
BPR-17	*B. amyloliquefaciens*	Soil and vegetation	148,296

* Generated by Westerdijk Fungal Biodiversity Institute, Utrecht, The Netherlands [26]; ** 100% Identical match to *B. velenesis* by 16 s sequencing.

**Table 2:** MIC values against various pathogens (µg/mL).

Strain	*C. perfringens*	*E. coli*	*P. aeruginosa*	*S. aureus*	*S. enterica*
BPR-11 EtOAc	100	100	NA	100	NA
BPR-16 EtOAc	50	50	100	50	100
BPR-17 EtOAc	50	50	100	50	100
F1 EtOAc	25	25	50	25	50
BPR-11 crude	NA	NA	NA	NA	NA
BPR-16 crude	NA	NA	NA	NA	NA
BPR-17 crude	NA	NA	NA	NA	NA
F1 crude	NA	NA	NA	NA	NA

NA: not active = No signs of inhibition detected.

**Table 3:** Selected ion monitoring channels from total ion chromatography to record specific ions at defined *m*/*z*.

Target (Analyte)	Molecular Weight	Scan Range (*m*/*z*)	Quantitative Ion (*m*/*z*)
C_13_ surfactin C	1008.3	100–1500	1007.3
C_14_ surfactin C	1022.3	100–1500	1021.3
C_15_ surfactin C	1036.3	100–1500	1035.3
plemeC_16_ surfactin C	1050.3	100–1500	1049.3

**Table 4:** Analysis value and half-life of C_13_ to C_16_ surfactin C when incubated with microsomes.

Target (Analyte)	Molecular Weight	k	t_1/2_ (Mins)
C_13_ surfactin C	1008.3	0.0006	1155
C_14_ surfactin C	1022.3	0.0018	378
C_15_ surfactin C	1036.3	0.0008	866.25
C_16_ surfactin C	1050.3	0.0007	990
